# Light Modulates Leptin and Ghrelin in Sleep-Restricted Adults

**DOI:** 10.1155/2012/530726

**Published:** 2012-08-14

**Authors:** Mariana G. Figueiro, Barbara Plitnick, Mark S. Rea

**Affiliations:** Lighting Research Center, Rensselaer Polytechnic Institute, 21 Union Street, Troy, NY 12180-3352, USA

## Abstract

Acute and chronic sleep restrictions cause a reduction in leptin and an increase in ghrelin, both of which are associated with hunger. Given that light/dark patterns are closely tied to sleep/wake patterns, we compared, in a within-subjects study, the impact of morning light exposures (60 lux of 633-nm [red], 532-nm [green], or 475-nm [blue] lights) to dim light exposures on leptin and ghrelin concentrations after subjects experienced 5 consecutive days of both an 8-hour (baseline) and a 5-hour sleep-restricted schedule. In morning dim light, 5-hour sleep restriction significantly reduced leptin concentrations compared to the baseline, 8-hour sleep/dim-light condition (*t*_1,32_ = 2.9; *P* = 0.007). Compared to the 5-hour sleep/dim-light condition, the red, green, and blue morning light exposures significantly increased leptin concentrations (*t*_1,32_ = 5.7; *P* < 0.0001, *t*_1,32_ = 3.6; *P* = 0.001, and *t*_1,32_ = 3.0; *P* = 0.005, resp.). Morning red light and green light exposures significantly decreased ghrelin concentrations (*t*_1,32_ = 3.3; *P* < 0.003 and *t*_1,32_ = 2.2; *P* = 0.04, resp.), but morning blue light exposures did not. This study is the first to demonstrate that morning light can modulate leptin and ghrelin concentrations, which could have an impact on reducing hunger that accompanies sleep deprivation.

## 1. Introduction

Leptin is a hormone secreted from the white adipocytes and plays a role in the maintenance of energy homeostasis. Ghrelin is a 28-amino acid peptide hormone secreted by the stomach. Although the role of ghrelin in energy homeostasis is still unclear, its secretion depends largely on nutritional state, with ghrelin concentrations increasing prior to a meal and decreasing after eating [1].

Both chronic and acute sleep restriction cause a reduction in serum concentrations of leptin and an increase in serum ghrelin concentrations [2–7]. Subjects who had a 4-hour sleep opportunity for 6 consecutive nights had 19% lower mean and 26% lower maximum concentrations of leptin [2]. Taheri et al. [4] showed that those subjects who habitually slept 5 hours compared to 8 hours per night had 15.5% lower leptin concentrations. Sleep restriction is also associated with an increased sense of hunger [3, 7].

Leptin concentrations exhibit a strong circadian pattern, with the highest concentrations occurring at night and the lowest concentrations in the middle of the day. It has been shown that neither feeding time nor adrenalectomy affected the rhythmicity of leptin release. Ablation of the suprachiasmatic nuclei (SCN) in rats, where the circadian pacemaker is located, however, eliminated leptin circadian rhythmicity in rodents, suggesting that the central circadian clock regulates leptin expression [8]. Ghrelin concentrations also exhibit a strong circadian pattern, with the highest concentrations occurring during the daytime hours and the lowest concentrations occurring at night [9].

Light has an impact on hormone production through stimulation of the SCN. Light/dark patterns, conveyed from the retina to the SCN via the retinohypothalamic (RHT) tract, are the major synchronizer of the SCN to the 24-hour solar day. The SCN controls the timing of production of a series of biomarkers, such as the timing of melatonin synthesis. Melatonin, a hormone produced at night and in darkness, is believed to be a primary timing messenger for the entire body, providing seasonal and daily photoperiod information to every neural, physiological, and cellular system. Nighttime exposures to certain light levels and spectra will cease or diminish the production of melatonin. In humans, nocturnal melatonin suppression is maximally sensitive to short-wavelength (blue) light peaking close to 460 nanometers (nm). Light can also impact hormones and brain activity without affecting nocturnal melatonin production [10, 11]. Figueiro and Rea [11] showed that exposures to both 470-nm (blue) and 630-nm (red) lights increased cortisol concentrations at night, while only blue light suppressed melatonin. In another study, Figueiro et al. [10] demonstrated that blue and red light exposures at night increased alertness, measured via electroencephalogram, showing that melatonin suppression is not required for light-induced nighttime alertness.

Fluctuations in body weight have been associated with changes in day lengths, suggesting a role of the biological clock in regulating metabolism. It has been shown that in middle-aged rats, daily melatonin administration (liquid diet containing 0.2 mcg/mL melatonin) decreased weight gain in response to a high-fat diet and decreased nighttime plasma leptin concentrations within 3 weeks of the treatment [12]. These effects were independent of total food consumption. Thus, it seems that the circadian clock may play an important role in determining body weight, likely by influencing the expression and secretion of hormones, such as melatonin.

The present study was an investigation of how different light spectra might counteract the impact of sleep restriction on concentrations of leptin and ghrelin in subjects who maintained a 5-hour sleep schedule for 5 consecutive days. Since sleep restriction decreases leptin and increases ghrelin concentrations and since light has a strong influence on hormone production, such as melatonin production, it was conceivable to hypothesize that light modulate concentrations of leptin and ghrelin after sleep restriction. Specifically, as in previous studies, it was hypothesized that, after sleep restriction, morning leptin concentrations collected in dim light would be lower and ghrelin concentrations would be higher than after the baseline week (8-hour sleep opportunity). It was further hypothesized that when subjects underwent a sleep-restricted schedule (5-hour sleep opportunity per night for 5 consecutive nights), exposure to narrowband long-wavelength (red), middle-wavelength (green), and short-wavelength (blue) lights would counteract the effect of sleep deprivation on these biomarkers by increasing concentrations of plasma leptin and decreasing concentrations of plasma ghrelin. If it could be shown that narrowband lights modulate concentrations of leptin and ghrelin, a secondary goal of the study was to determine whether their spectral sensitivities were similar to that of acute melatonin suppression, which is maximally sensitive to short-wavelength light.

## 2. Methods

### 2.1. Subjects

Eleven subjects (six females) participated in the experiment. The mean ± standard deviation (SD) age of participants was 27.4 ± 8.7 years and the mean ± SD body mass index (BMI) was 25.2 ± 4.2 kg/m^2^. All subjects included in the study were screened for medical illness and previous gastrointestinal surgery, and all reported being nonsmokers and medication-free. Shift workers or subjects who had traveled across time zones four weeks prior to the experiment were not accepted into the study. The Institutional Review Board at Rensselaer Polytechnic Institute approved the study and participants provided informed consent and were paid for their participation.

### 2.2. Lighting Conditions

Subjects experienced four morning light-exposure conditions for two hours upon wakening: (1) 60 lux of red light (0.325 W/m^2^, peak = 633 nm, full-width-half-maximum [FWHM] = 14 nm); (2) 60 lux of green light (0.105 W/m^2^, peak = 532 nm, FWHM = 34 nm); (3) 60 lux of blue light (0.585 W/m^2^, peak = 475 nm, FWHM = 21 nm); (4) dim light conditions (<0.5 lux at the eye of a 630-nm, red light). Two light emitting diodes (LEDs) were mounted to each of the goggle lenses. Prior to every experiment, the light goggles were calibrated in the laboratory using a spectroradiometer (model 2300i, Action Research, Ottobrunn, Germany and Spectra-Sense Version 4.3.0, Advanced Fiber Sensors Inc., Ann Arbor, MI, USA) with a UV-VIS optical fiber ending in a Lambertian diffuser.

### 2.3. Procedures

The experiment was conducted over 9 weeks; data were collected in the laboratory on alternate weeks starting at the end of week 1 (Thursday evening). Participants were required to maintain an 8-hour sleep schedule during the first baseline week. During weeks 3, 5, 7, and 9, participants were asked to maintain a 5-hour sleep schedule from Sunday through Thursday prior to data collection. During the sleep restricted weeks, subjects were asked to maintain the same wake-up times as the baseline week and they were required to go to bed 3 hours later than their bedtimes during the baseline week. Six subjects (group 1) were asked go to bed at 10:00 p.m. and wake up at 6:00 a.m. and five subjects (group 2) were asked to go to bed at 11:00 p.m. and wake up at 7:00 a.m. during the 8-hour schedule weeks. The two groups were selected based on their typical wake-up times, which were always kept the same during the experiment. During the sleep-restricted schedule weeks, the same six subjects who had a wake-up time at 6:00 a.m. were required to stay awake until 1:00 a.m. and the other five subjects who had a wake-up time at 7:00 a.m. were required to go to bed at 2:00 a.m. During weeks 1, 3, 5, 7, and 9 subjects were asked to sleep at the laboratory on Thursday evenings, and data collection occurred on Friday mornings. During weeks 2, 4, 6, and 8, participants were required to maintain a regular 8-hour sleep schedule (similar to the baseline week) so that they could return to baseline; they were not required to come to the lab at the end of those weeks.

No naps were allowed throughout the experiment. Compliance was assured from data obtained from actigraphy and checked against their sleep log reports. Participants wore a wrist Dimesimeter during the length of the experiment. The Dimesimeter is a calibrated light meter that contains accelerometers and three sensors which record and store data in flash memory [13]. Light data from the photosensor channels were downloaded to a host computer and converted into photopic illuminance, circadian light (CL_A_), and circadian stimulus (CS) levels. Concisely, photopic illuminance is irradiance transformed by the photopic luminous efficiency function (*V*_(*λ*)_), providing an orthodox measure of the spectral sensitivity of the human fovea. CL_A_, based on nocturnal melatonin suppression, is irradiance weighted by the spectral sensitivity of the retinal phototransduction mechanisms stimulating the SCN. CS is a transform of CL_A_ into relative units from 0, the threshold for circadian system activation, to 0.7, response saturation, and corresponds to relative suppression of nocturnal melatonin after one hour of light exposure for a 2.3 mm diameter pupil during the midpoint of melatonin production.

Participants reported for the first, baseline overnight session at 9:00 p.m. (group 1) and at 10:00 p.m. (group 2) and were given an 8-hour sleep opportunity from 10:00 p.m. to 6:00 a.m. (group 1) and from 11:00 p.m. to 7:00 a.m. (group 2). During weeks 3, 5, 7, and 9, participants reported to the laboratory at 11:00 p.m. (group 1) and midnight (group 2) and were kept awake until their scheduled bedtimes, after which they were given a 5-hour sleep opportunity in complete darkness. Again, rest/activity patterns were analyzed and confirmed for compliance to the schedule. Participants were not allowed to eat or drink from 12 hours prior to awakening until the last blood draw was completed on the following morning. Caffeine and alcohol use was voluntarily restricted starting 24 hours prior to the overnight session in the laboratory.

For the baseline, 8-hour sleep condition (week 1) and for the first 5-hour sleep-restricted condition (week 3), participants were in dim red light (peak *λ* = 630 nm; less than 0.5 lux at the eye) for 120 minutes after morning awakening in the laboratory. For weeks 5, 7, and 9 participants were exposed to the red, green, or blue lights for 120 minutes upon awakening; light spectrum (red, green, and blue lights) presentation was counterbalanced across subjects.

For every morning session, the first blood sample was collected 60 minutes after awakening and subsequent samples were collected 90 and 120 minutes after awakening. Blood was drawn into two 4 mL vacutainer tubes containing K2 EDTA 7.2 mg, and immediately centrifuged for 10 minutes at 3500 rpm at 4°C. The plasma was separated and frozen at −20°C until assayed. This protocol was followed for every laboratory session.

## 3. Data Analyses

Plasma leptin and total ghrelin concentrations were measured in duplicate by radioimmunoassay using commercially available kits from Millipore (Billerica, MA, USA). The lower limits of detection were 0.75 ng/mL for leptin and 93 pg/mL for ghrelin. Intra- and inter-assay precisions were 5% and 7% for leptin and 6% and 16% for ghrelin, respectively. All samples from each overnight session were processed in the same batch.

Five (sleep-duration/light-exposure combinations) by 3 (sample times) analyses of variance (ANOVAs) were performed on both plasma leptin and ghrelin concentrations. Post hoc, two-tailed Student's *t*-tests were performed to further investigate the significant main effects and interactions from the ANOVAs using the unnormalized data.

In addition, plasma leptin and ghrelin concentrations following the 5-hour sleep restriction in the dim-light condition were taken as the reference values for post hoc inferential statistical analyses. Plasma leptin and ghrelin concentrations at 60, 90, and 120 minutes after 8-hour sleep/dim-light condition and concentrations after light exposures (red, green, or blue light) were normalized (ratio) to concentrations at 60, 90, and 120 minutes after the 5-hour sleep/dim-light condition. One-sample, two-tailed *t-*tests using the data collected at the three sample times were used to determine whether the changes in leptin and ghrelin concentrations for each of the other four experimental conditions were significantly different than those obtained under the 5-hour sleep/dim-light condition. An a *priori* criterion probability for a Type I error was set at 0.05 for all post hoc statistical analyses.

## 4. Results

The ANOVA using leptin concentrations revealed significant main effects of sleep-duration/light-exposure combinations (*F*_4,40_ = 3.3; *P* = 0.02) and of sample times (*F*_2,20_ = 7.8; *P* = 0.003). Post hoc, two-tailed Student's *t-*tests revealed that leptin concentrations after the morning red light exposure were significantly greater than after the 8-hour sleep/dim-light condition (*P* = 0.009) and after the 5-hour sleep/dim-light condition (*P* = 0.001). The mean ± standard error of the mean (SEM) and the median concentrations are presented in Table 1.

The ANOVA using ghrelin concentrations revealed a significant main effect of sample times (*F*_2,20_ = 17.5; *P* < 0.0001) and an almost significant interaction between sleep-duration/light-exposure combinations and sample times (*F*_8,80_ = 1.8; *P* = 0.09). Ghrelin concentrations increased significantly over the course of the two-hour experiment. The mean ± SEM ghrelin concentrations were 927 ± 69 pg/mL after 60 minutes, 947 ± 73 pg/mL after 90 minutes, and 961 ± 72 pg/mL after 120 minutes. Although the main effect of sleep-duration/light-exposure combinations was not statistically significant (*F*_4,40_ = 1.6; *P* = 0.2), ghrelin concentrations were the highest after the 5-hour sleep/dim-light condition and all three morning light exposure spectra reduced ghrelin concentrations. The mean ± SEM and median ghrelin concentrations are listed in Table 1.

In order to determine the changes in leptin and ghrelin concentrations by light relative to the reference condition (5-hour sleep/dim-light condition), data collected at 60, 90, and 120 minutes for the other four experimental conditions (8-hour sleep/dim-light, 5-hour sleep/red-light condition, 5-hour sleep/green-light condition, and 5-hour sleep/blue-light condition) were normalized to the data collected at 60, 90, and 120 minutes during the 5-hour sleep/dim-light condition.

**Figure 1:**
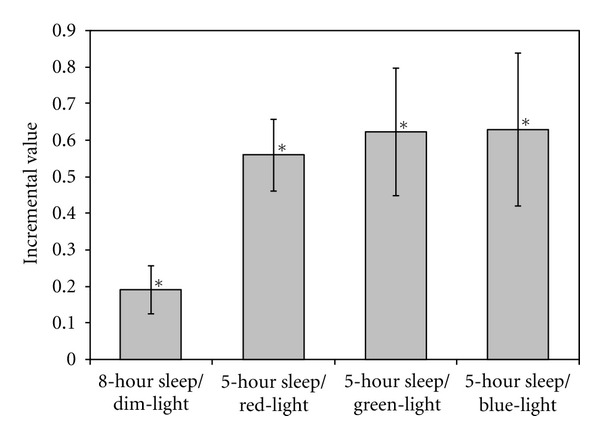
Differences (incremental values) in the normalized leptin concentrations (mean ± SEM) relative to the 5-hour sleep/dim-light condition. Sample concentrations collected at 60, 90, and 120 minutes for the 8-hour sleep/dim-light condition, those for the 5-hour sleep/red-light condition, those for the 5-hour sleep/green-light condition, and those for the 5-hour sleep/blue-light condition were each normalized to the values obtained at 60, 90, and 120 minutes for the reference, 5-hour sleep/dim-light condition. These normalized values were averaged across subjects and times. The incremental values reflect the difference in the averages of the four different sleep/lighting conditions from the 5-hour sleep/dim-light condition. Incremental values after the 8-hour sleep/dim-light condition and after all three lighting conditions were significantly (*P* < 0.05) greater than those in the reference, 5-hour sleep/dim-light condition.

One-sample two-tailed *t-*tests revealed that, with respect to the 5-hour sleep/dim-light condition, normalized leptin concentrations (average of the three samples collected at 60, 90, and 120 minutes) were significantly higher for the 8-hour sleep/dim-light condition (*t*_1,32_ = 2.9; *P* = 0.007) and for the (sleep-restricted) red-light (*t*_1,32_ = 5.7; *P* < 0.0001), green-light (*t*_1,32_ = 3.6; *P* = 0.001), and blue-light conditions (*t*_1,32_ = 3.0; *P* = 0.005). Leptin concentrations following the 8-hour sleep condition were nearly 20% higher than those following the 5-hour sleep condition, both of which were obtained under dim light. Exposure to the three morning light spectra increased leptin concentrations by more than 50% relative to the reference sleep-restricted/dim-light condition.  Figure 1 illustrates these effects.

**Figure 2:**
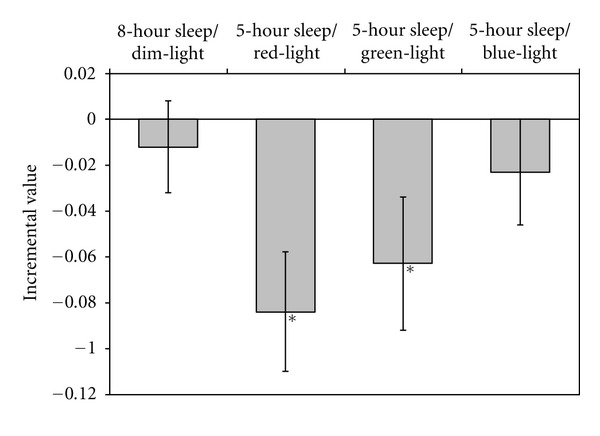
Differences (incremental values) in the normalized ghrelin concentrations (mean ± SEM) relative to the reference, 5-hour sleep/dim-light condition. Sample concentrations collected at 60, 90, and 120 minutes for the 8-hour sleep/dim-light condition, those for the 5-hour sleep/red-light condition, those for the 5-hour sleep/green-light condition, and those for the 5-hour sleep/blue-light condition were each normalized to the values obtained at 60, 90, and 120 minutes for the reference, 5-hour sleep/dim-light condition. These normalized values were averaged across subjects and times. The incremental values reflect the difference in the averages of the four different sleep/lighting conditions from the 5-hour sleep/dim-light condition. Incremental values after the 5-hour sleep/red-light and 5-hour sleep/green-light conditions were significantly lower than those in the reference, 5-hour sleep/dim-light condition.

One-sample two-tailed *t-*tests revealed that compared to the 5-hour sleep/dim-light condition, the normalized ghrelin concentrations (average of the three sample concentrations collected at 60, 90, and 120 minutes) were significantly lower after exposure to morning red (*t*_1,32_ = 3.3; *P* < 0.003) and green (*t*_1,32_ = 2.2; *P* = 0.04) lights, but not following the blue-light exposure (*t*_1,32_ = 1.0; *P* = 0.3) and not after the 8-hour sleep/dim-light condition (*t*_1,32_ = 0.6; *P* = 0.6).  Figure 2 illustrates these effects.

## 5. Discussion

The present study is the first to demonstrate that narrowband, morning light exposures can modulate leptin and ghrelin concentrations, biomarkers associated with hunger, following sleep restriction. Previous studies have shown that sleep deprivation decreased overall leptin concentrations by 18% and increased ghrelin concentrations by 28% [2, 3, 7]. The present results are consistent with the literature in that they reveal a decrease in leptin concentrations of 19% when subjects were sleep restricted. Although ghrelin concentrations following sleep restriction were lower than following the 8-hour sleep/dim-light condition in the present study, these reductions were not statistically significant.

The present results extend those in the literature by showing that light exposure in the morning can increase leptin and decrease ghrelin concentrations in sleep-restricted individuals although, again, the impact of morning light on leptin concentrations was more robust than on ghrelin concentrations. It is possible, however, that still higher light levels (e.g., morning daylight) are needed to reduce ghrelin concentrations. An experimental protocol similar to the one used here should be conducted to examine this possibility.

It seems unlikely that the increases in leptin concentrations observed in the present study were the result of a circadian phase shift due to subjects' delayed bedtimes [14]. Peak concentrations of leptin occur during the middle of the night, but leptin concentrations following the 5-hour sleep/dim-light condition were significantly lower than those in the 8-hour sleep/dim-light condition (and for all of the morning light exposure conditions). If a light-induced phase shift of the leptin rhythm had occurred before sleep during the later bedtimes, leptin concentrations in the morning would have been higher, not lower as observed. Moreover, the light measurements from the wrist actigraph showed that the median light levels between 10:00 p.m. and the participants' bedtimes were below 12 lux of “white” light, which would result in a predicted melatonin suppression of less than 1%, suggesting that these evening light levels were too low to delay their circadian clock [15, 16].

The pathways are not known through which light might modulate leptin and ghrelin concentrations. Since it has been shown that rats on a high-fat diet and treated with melatonin had a decrease in leptin concentrations compared to controls [12], the impact of light on leptin concentrations could have been mediated by light's ability to suppress morning melatonin. In the present study, melatonin concentrations were not measured, so it is not possible to make a direct link between the observed effects of light on leptin and ghrelin and melatonin concentrations. Although it cannot be ruled out that light-induced melatonin suppression also may play a role in mediating these effects as shown in animal models [12], the present study logically suggests that light-induced increases in leptin concentrations and decreases in ghrelin concentrations were not dependent upon acute melatonin suppression. The level of morning red light exposure used in this study was not high enough to suppress melatonin [10], but, like the blue- (and green-) light exposure, it was sufficient to modulate leptin concentrations. The same, or a parallel, effect may have been observed by Figueiro and colleagues [10, 11]. They showed that the same level of red light used in the present study increased cortisol concentrations and alertness, as measured by electroencephalogram [10, 11]. Possibly the same neural mechanisms mediating the observed light-induced alertness and observed increase in cortisol concentrations are also mediating the effects obtained in this study.

Although we cannot determine the spectral sensitivities of light-induced leptin and ghrelin modulation in this experiment, the fact that the morning red light had a significant impact on both leptin and ghrelin concentrations strongly suggests that the long-wavelength cones contribute to these acute responses; there is no other known photoreceptor in the retina that would have sufficient sensitivity to red light to evoke the observed effects. A more extensive study should be performed to determine the spectral sensitivities of the light-induced increases in leptin and decreases in ghrelin before we can conclude that this is a cone response.

Moreover, it is not known if color information relayed to the brain, rather than retinal irradiance levels, per se, is mediating these effects. Studies suggest that colors seen as “warm” (red, orange, yellow) evoke feelings of arousal while colors seen as “cool” (violet, blue, green) are associated with calming feelings. The color red, for example, has been associated with feelings of danger, love, rage, and excitement as well as negative feelings such as anxiety, anger, and annoyance [17–22]. It has been suggested that the color red increases human receptiveness to external stimuli and increases excitation, therefore affecting a person's emotional and motor responses [17–22]. It is conceivable that these subjective interpretations of color may be mediating the physiological changes in leptin and ghrelin concentrations.

The present results need to be replicated in a larger group of subjects, but they suggest that light can have a significant impact on reducing hunger that accompanies sleep deprivation. It has been shown that obese individuals have higher leptin concentrations than lean individuals and are more resistant, or have a greater tolerance, to the effects of leptin. Animal studies should be performed to determine the pathways via which light is modulating these biomarkers and if light can change leptin resistance in obese animals. Whether light can be used as a nonpharmacological tool to clinically impact obesity should be the subject of future research.

## Figures and Tables

**Table 1:** Mean ± SEM and median concentrations of leptin and ghrelin. Exposure to red light significantly (*P* < 0.05) increased leptin concentrations compared to the remaining in dim-light condition after both 8-hour sleep and 5-hour sleep schedules.

	Leptin		Ghrelin	
	Mean ± SEM (ng/mL)	Median (ng/mL)	Mean ± SEM (pg/mL)	Median (ng/mL)
8-hour sleep/dim	9.7 ± 2.3	7.9	979 ± 87	945
5-hour sleep/dim	9.5 ± 2.6	6.2	983 ± 70	957
5-hour sleep/green	11.6 ± 2.7	8.4	894 ± 43	847
5-hour sleep/blue	11.5 ± 2.6	9.1	962 ± 88	884
5-hour sleep/red	12.0 ± 2.7*	9.8	906 ± 90	768

*P = 0.05.
